# hsa_circ_0056856 in the serum serves as a potential novel biomarker for disease activity in psoriasis

**DOI:** 10.1097/CM9.0000000000002166

**Published:** 2022-08-10

**Authors:** Mingwang Zhang, Minna Han, Jian Li, Kang Tao, Xin Yang, Haihao Zheng, Rongya Yang, Zhifang Zhai

**Affiliations:** 1 Department of Dermatology, Southwest Hospital, Army Medical University, Chongqing 400038, China; 2 Department of Dermatology, The Seventh Medical Center of People's Liberation Army General Hospital, Beijing 100010, China; 3 Southern Medical Branch of People's Liberation Army General Hospital, Beijing 100000, China; 4 Department of Dermatology, Yanbian University Hospital, Yanji, Jilin 133000, China.

*To the Editor*: Psoriasis is a chronic immune-related inflammatory skin disease. The clinical severity of psoriasis varies greatly, and recurrence occurs frequently.^[1]^ Many attempts have been made to identify biomarkers for psoriasis; however, there are currently no specific markers that can accurately monitor disease severity and treatment efficacy. Circular RNAs (circRNAs) are a novel class of endogenous noncoding RNAs that are highly stable and resistant to RNase R since they can form covalently closed loops without 5′ caps and 3′ tails.^[2]^ It has been reported that circRNAs could exist in body fluids, such as serum and plasma, and could serve as molecular biomarkers in the diagnosis and prognosis of several diseases.^[3]^ In this study, we detected the alteration of circRNAs and investigated the potential value of hsa_circ_0056856 as a biomarker for disease severity in serum samples from psoriasis patients and healthy controls (HCs).

A total of 125 patients diagnosed with psoriasis vulgaris were recruited from the Seventh Medical Center of PLA General Hospital. We collected all the patient serum samples and 20 patient peripheral blood mononuclear cell (PBMC) samples from the same cohort at first admission to the hospital. We also obtained 30 patient serum samples after treatment for 7 to 8 weeks. Disease severity was assessed by the psoriasis area and severity index (PASI). None of the patients had other autoimmune diseases or were on systemic treatment at least 1 month prior to study participation. Serum samples from 75 healthy subjects, whose age and sex were matched with the psoriasis group, were collected at the Medical Examination Center of the same hospital. Details of all samples are summarized in Supplementary Table 1. This study was conducted in adherence with the principles of the *Declaration of Helsinki* and was approved by the Ethics Committee of the Seventh Medical Center of PLA General Hospital (No. 2020-084). Written informed consent was obtained from all participants.

First, we performed a circRNA microarray to identify the expression patterns of circRNAs in serum from five psoriasis patients and five normal controls. A total of 11,582 circRNAs were detected, and a box plot showed a similar distribution of log2 ratios among the ten samples after quantile normalization [Supplementary Figure 1A]. The genomic locations of their source genes were exonic (9741, 84.10%), intronic (798, 6.89%), sense overlapping (751, 6.48%), antisense overlapping (205, 1.77%), and intergenic (87, 0.75%) [Supplementary Figure 1B]. Approximately 66.32% (7681/11,582) of the identified circRNAs were >2k bp in length [Supplementary Figure 1C], and the chromosome distribution of the circRNAs showed that chromosome 1 had the most (1165), while chromosome 21 had the fewest (123) [Supplementary Figure 1D].

To explore the differentially expressed (DE) profiles of serum circRNAs in psoriasis, hierarchical clustering analysis was employed, and the results showed that the circRNA expression was significantly changed between psoriasis patients and HCs [Supplementary Figure 2A]. A total of 132 circRNAs were significantly changed (|log2 (fold change)| > 1 and *P* < 0.05) in serum from psoriasis patients, with 123 upregulated and nine downregulated circRNAs, compared with HCs [Supplementary Figure 2B and Supplementary Table 2]. Among them, the most upregulated circRNA was hsa_circ_0056856, and the circRNA showing the greatest downregulation was hsa_circ_0112550. Subsequently, we screened DE circRNAs based on the raw intensity, fold-change, and statistical value in the RNA-seq data. Ten DE circRNAs were selected for further analysis by real-time reverse transcription quantitative polymerase chain reaction (RT-qPCR) in serum samples from 20 psoriasis patients and 20 HCs. All primers were synthesized by Sangon Biotech (Shanghai, China) and are listed in Supplementary Table 3. The results showed that six circRNAs, including hsa_circ_0056856 (*U* = 400, *Z* = 5.41, *P* < 0.001) (Mann-Whitney *U* test, *U*-value represents the difference of rank-sum and *Z* score means the difference of median between these two groups), hsa_circ_0023461 (*U* = 286, *Z* = 2.326, *P* = 0.02), hsa_circ_0082326 (*U* = 291, *Z* = 2.462, *P* = 0.013), hsa_circ_0001255 (*U* = 298, *Z* = 2.651, *P* = 0.007), hsa_circ_0055440 (*U* = 282, *Z* = 2.218, *P *= 0.026), and hsa_circ_0112550 (*U* = 117, *Z* = −2.245, *P* = 0.024), were consistent with the RNA-seq data, among which hsa_circ_0056856 exhibited the most significant changes and was finally selected for further validation [Figure 1A].

**Figure 1:**
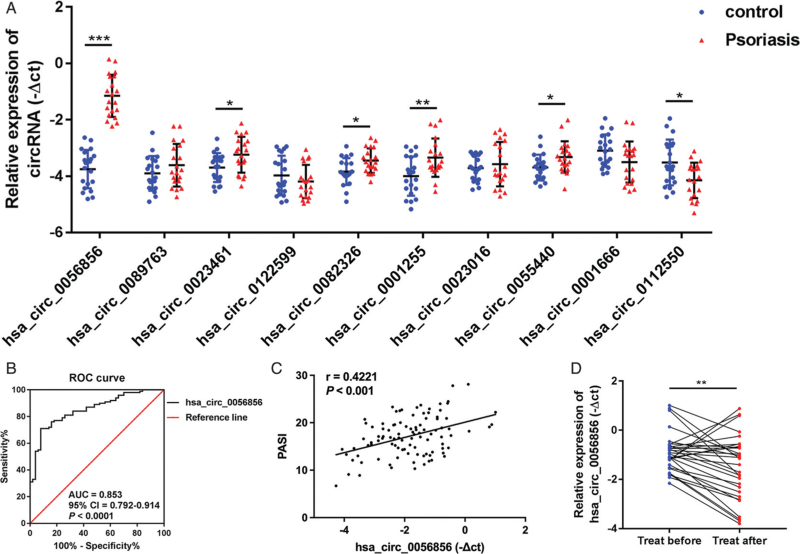
hsa_circ_0056856 as a biomarker for disease severity in psoriasis. (A) The expression profiles of selected circRNAs in serum between 20 psoriasis patients and 20 HCs by RT-qPCR validation. (B) The ROC curve of hsa_circ_0056856 was calculated based on the −ΔCt value in serum from 100 psoriasis patients and 50 HCs. (C) Correlation between serum levels of hsa_circ_0056856 and PASI scores in 100 psoriasis patients. (D) hsa_circ_0056856 expression was measured before and after 7 to 8 weeks of treatment in 30 psoriasis patients by RT-qPCR. ^∗^*P* < 0.05; ^∗∗^*P* < 0.01; ^∗∗∗^*P* < 0.001. circRNAs: Circular RNAs; HCs: Healthy controls; PASI: Psoriasis area and severity index; ROC: Receiver operating characteristic; RT-qPCR: Real-time reverse transcription quantitative polymerase chain reaction.

To explore the diagnostic value of hsa_circ_0056856 as a biomarker for psoriasis, we analyzed the expression of hsa_circ_0056856 in a larger cohort of serum samples, including 100 psoriasis patients and 50 HCs. In addition, PBMC samples from 30 patients and 20 controls were also collected from the same cohort [Supplementary Table 1]. Consistent with the above findings, hsa_circ_0056856 was dramatically increased in both psoriasis patients’ serum (*U* = 735, *Z* = 7.037, *P* < 0.001) and PBMCs (*U* = 106, *Z* = 3.842, *P* < 0.001) compared with HCs [Supplementary Figure 3A, B]. Moreover, receiver operating characteristic curve analysis showed that the area under curve value of hsa_circ_0056856 was 0.853 (95% CI = 0.792–0.914, *P* < 0.0001) (Wilson/Brown method) [Figure 1B]. These results suggested that hsa_circ_0056856 could be a potential biomarker for psoriasis diagnosis.

Next, we evaluated the potential value of hsa_circ_0056856 as a biomarker for disease severity in psoriasis. We measured the correlation between the expression of hsa_circ_0056856 and the PASI score. Our results identified that the hsa_circ_0056856 levels in serum were positively correlated with PASI scores in psoriasis patients (*r* = 0.422, *P* < 0.0001) (Spearman's rank correlation coefficient) [Figure 1C]. In addition, we detected the expression of hsa_circ_0056856 in serum obtained from 30 patients at baseline and after 7 to 8 weeks of treatment. All patients achieved a significant improvement in PASI score [Supplementary Table 1], and the expression of hsa_circ_0056856 exhibited a significant downregulation after treatment (*Z* = 2.725, *P* = 0.006) (Wilcoxon signed-rank test, *Z* score means the difference of median between these two groups) [Figure 1D].

Previous studies have found that circRNAs can indirectly regulate gene expression by serving as miRNA sponges. Therefore, we predicted the potential miRNAs that could bind to hsa_circ_0056856 using Arraystar's homemade miRNA target prediction software. The top five miRNA response elements were selected as follows: miR-1301-3p, miR-140-3p, miR-93-3p, miR-19b-3p, and miR-187-5p. Next, we analyzed the potential targets of the five miRNAs using three online websites (TargetScan, TarBase, and MicroT-CDS), and 43, 82, 52, 23, and 78 candidate genes were screened out. A hsa_circ_0056856-miRNA- target mRNA interaction network was constructed through a ceRNA mechanism using Cytoscape software [Supplementary Figure 4A]. To further understand the underlying molecular mechanism of hsa_circ_0056856, we performed GO biological process enrichment analysis to investigate the functions of these target genes. The results showed that the target mRNAs in the ceRNA network were mainly involved in the regulation of protein serine/threonine kinase activity, histone modification, covalent chromatin modification, and peptidyl-lysine modification [Supplementary Figure 4B]. These results indicate that hsa_circ_0056856 could participate in epigenetic regulation in psoriasis through a ceRNA mechanism. However, further functional verification is required in future studies.

Accumulating evidence supports the use of circRNAs as diagnostic and therapy response biomarkers of disease.^[4]^ Because circRNAs are constantly stable and specific in serum and serum is easier to isolate, transport, and preserve in the clinic, these circRNAs could be used as therapeutic response and diagnostic tools for various diseases as well as potential viable therapeutic targets.^[3]^ hsa_circ_0056856 expression has been reported in several cell types including Nhek, Mcf7, A549, and HMEC cells.^[5]^ In the present study, we revealed that hsa_circ_0056856 was significantly increased in serum from psoriasis patients compared with HCs and could serve as an ideal biomarker for psoriasis diagnosis, severity, and therapeutic response evaluation. Moreover, we identified that hsa_circ_0056856 may participate in the pathogenesis of psoriasis through a ceRNA mechanism based on bioinformatic analysis, which could indirectly regulate gene expression and affect the histone modification biological process.

## Funding

This work was supported by grants from the National Natural Science Foundation of China (Nos. 81773316 and 82002120) and the National Science Foundation of Chongqing, China (No. cstc2021jcyj-msxmX0140).

## Conflicts of interest

None.

## Supplementary Material

Supplemental Digital Content

## Supplementary Material

Supplemental Digital Content

## Supplementary Material

Supplemental Digital Content

## Supplementary Material

Supplemental Digital Content
