# Supplementary material for: hsa_circ_0056856 in the serum serves as a potential novel biomarker for disease activity in psoriasis
Source: Chin Med J (Engl). 2022 Aug 10;135(14):1759–61. doi: 10.1097/CM9.0000000000002166 (PMC9509088; doi:10.1097/CM9.0000000000002166)
Supplement: Supplemental Digital Content [file cm9-135-1759-s001.doc]

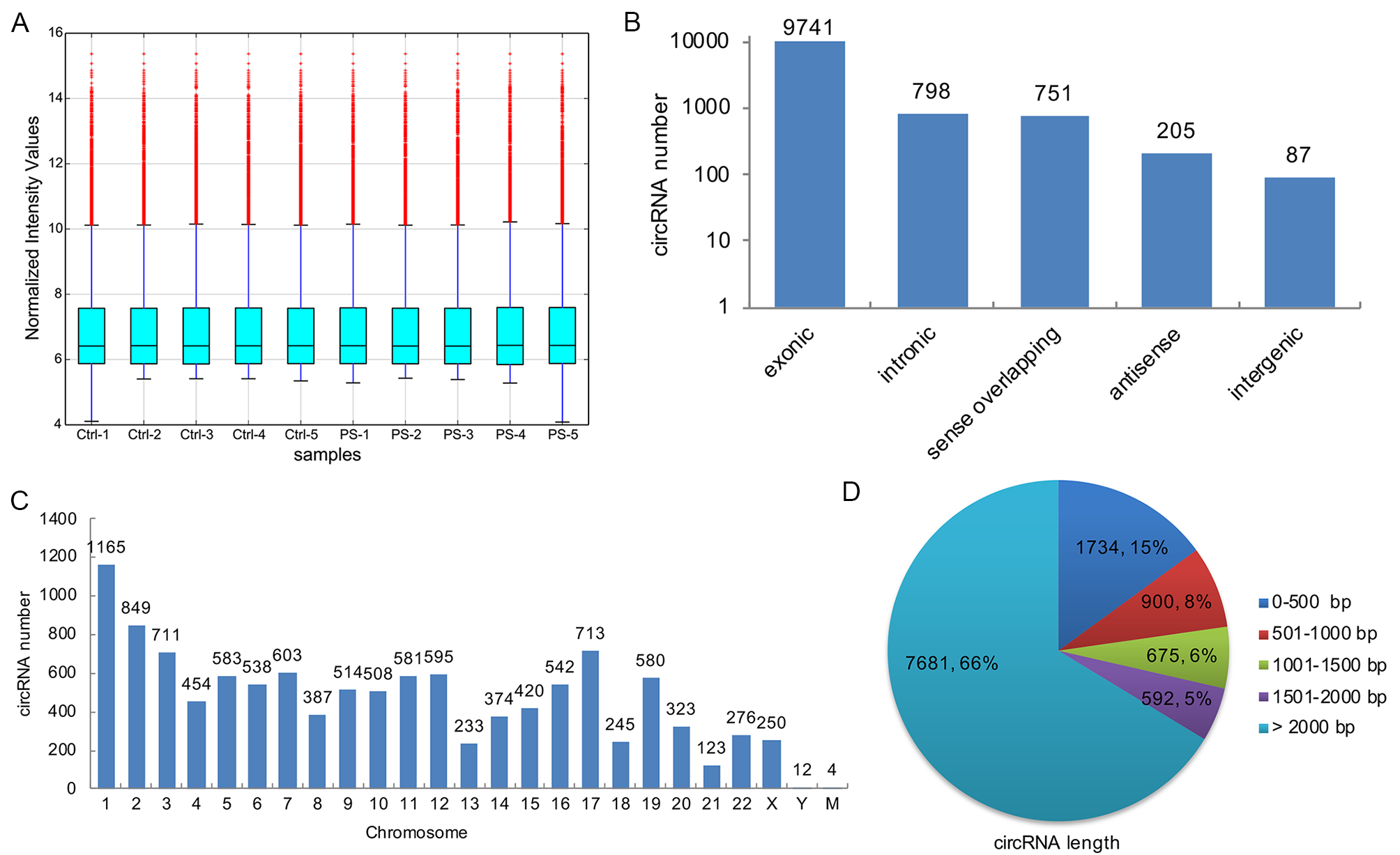


**Supplementary Figure 1:** Characteristics of circRNA expression profiles. (A) Box plot showing the expression levels of circRNAs in each sample. (B) The genomic position distribution of circRNAs. (C) The length distribution of circRNAs. (D) The distribution of circRNAs on chromosomes. circRNA: Circular RNA.

**
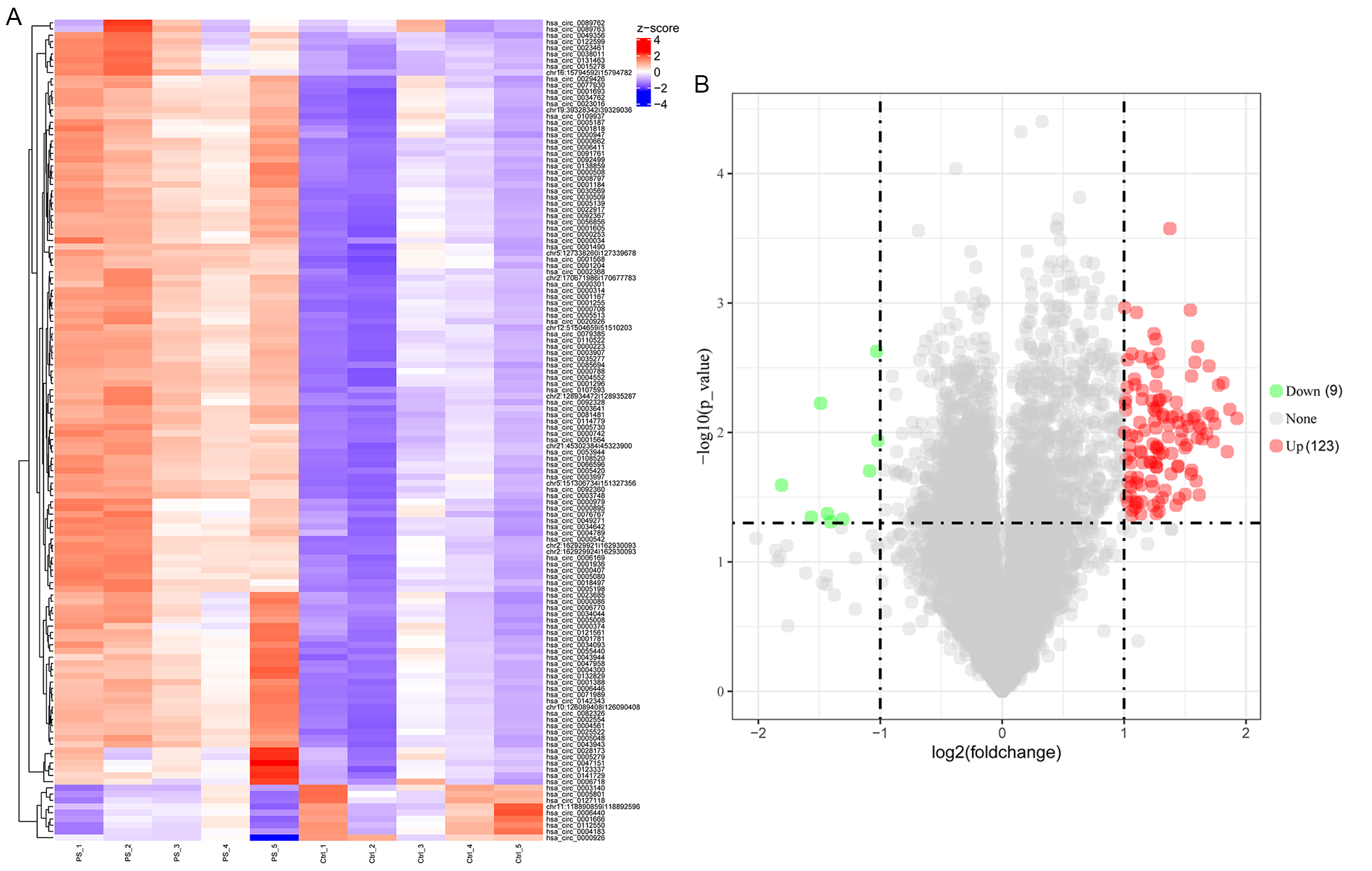
**

**Supplementary Figure 2:** Identification of DE circRNAs in psoriasis patients and HCs. (A) Hierarchical clustering analysis of DE circRNAs in psoriasis patients compared with HCs. Each column represents a sample, and each row represents a circRNA. Red indicates upregulation, and blue indicates downregulation. (B) Volcano plot of DE circRNAs in psoriasis patients compared with HCs. Upregulated circRNAs are indicated by red color, and downregulated circRNAs are indicated by green color. circRNAs: Circular RNAs; DE: Differentially expressed; HCs: Healthy controls.


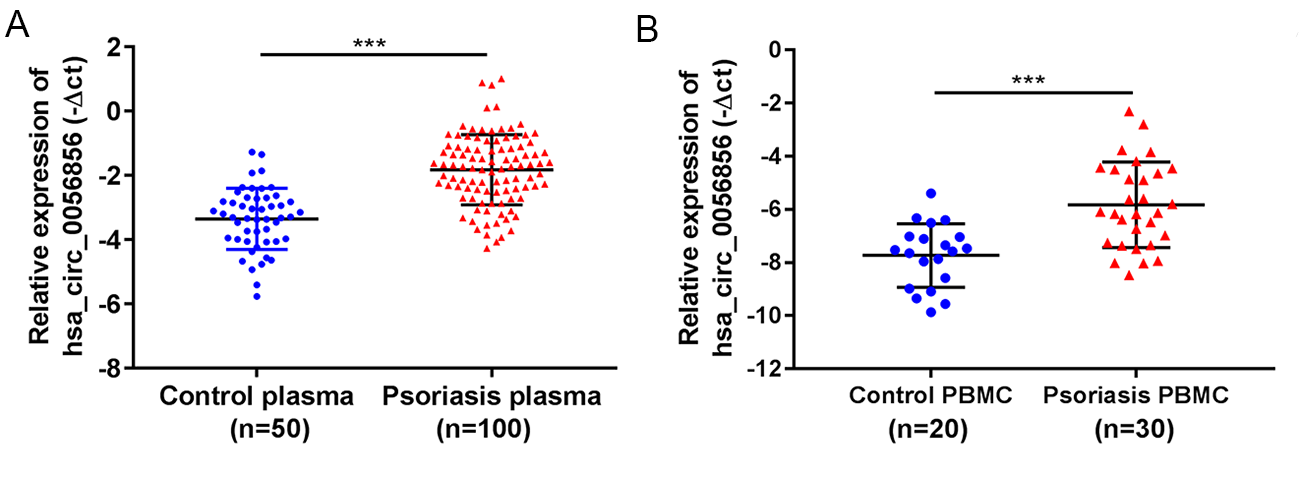


**Supplementary Figure 3:** circRNA expression signature for psoriasis diagnosis. (A) The expression of hsa_circ_0056856 in serum was detected in 100 psoriasis patients and 50 HCs by RT−qPCR. (B) The expression of hsa_circ_0056856 in PBMCs was detected in 30 psoriasis patients and 20 HCs by RT−qPCR. ^***^*P* < 0.001. circRNA: Circular RNA; HCs: Healthy controls; PBMCs: Peripheral blood mononuclear cells.


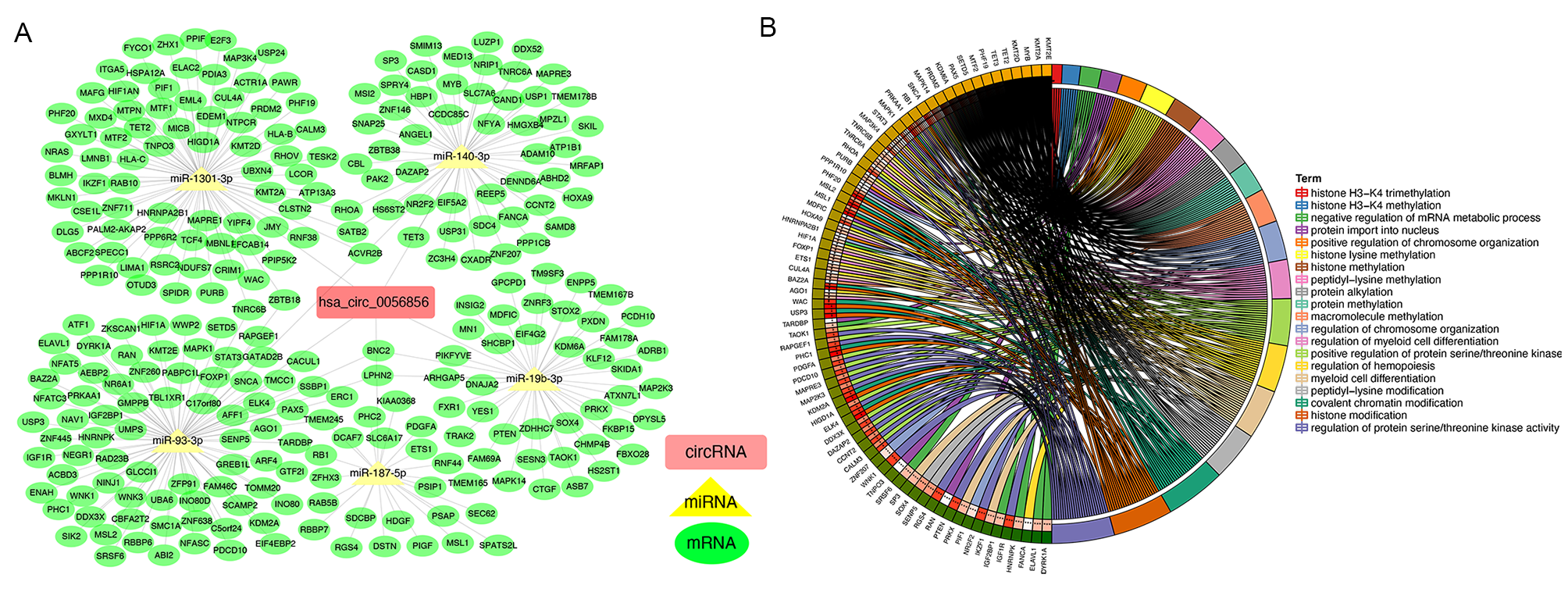


**Supplementary Figure 4:** Functional prediction of hsa_circ_0056856 in psoriasis. (A) Construction of the hsa_circ_0056856–miRNA–mRNA interaction network using Cytoscape. (B) The top 20 GO biological process terms of the target mRNAs based on the ceRNA network. circRNA: Circular RNA.
